# Molecular and cellular mechanisms underlying the antidepressant effects of ketamine enantiomers and its metabolites

**DOI:** 10.1038/s41398-019-0624-1

**Published:** 2019-11-07

**Authors:** Chun Yang, Jianjun Yang, Ailin Luo, Kenji Hashimoto

**Affiliations:** 1 Department of Anesthesiology, Tongji Hospital, Tongji Medical College, Huazhong University of Science and Technology, Wuhan, China; 2 Department of Anesthesiology, The First Affiliated Hospital of Zhengzhou University, Zhengzhou, China; 3 Division of Clinical Neuroscience, Chiba University Center for Forensic Mental Health, Chiba, Japan; 4 Present Address: Department of Anesthesiology and Perioperative Medicine, The First Affiliated Hospital of Nanjing Medical University, Nanjing, 210029 China

**Keywords:** Depression, Clinical pharmacology

## Abstract

Although the robust antidepressant effects of the *N*-methyl-d-aspartate receptor (NMDAR) antagonist ketamine in patients with treatment-resistant depression are beyond doubt, the precise molecular and cellular mechanisms underlying its antidepressant effects remain unknown. NMDAR inhibition and the subsequent α-amino-3-hydroxy-5-methyl-4-isoxazolepropionic acid receptor (AMPAR) activation are suggested to play a role in the antidepressant effects of ketamine. Although (*R*)-ketamine is a less potent NMDAR antagonist than (*S*)-ketamine, (*R*)-ketamine has shown more marked and longer-lasting antidepressant-like effects than (*S*)-ketamine in several animal models of depression. Furthermore, non-ketamine NMDAR antagonists do not exhibit robust ketamine-like antidepressant effects in patients with depression. These findings suggest that mechanisms other than NMDAR inhibition play a key role in the antidepressant effects of ketamine. Duman’s group demonstrated that the activation of mammalian target of rapamycin complex 1 (mTORC1) in the medial prefrontal cortex is reportedly involved in the antidepressant effects of ketamine. However, we reported that mTORC1 serves a role in the antidepressant effects of (*S*)-ketamine, but not of (*R*)-ketamine, and that extracellular signal-regulated kinase possibly underlie the antidepressant effects of (*R*)-ketamine. Several lines of evidence have demonstrated that brain-derived neurotrophic factor (BDNF) and its receptor, tyrosine kinase receptor B (TrkB), are crucial in the antidepressant effects of ketamine and its two enantiomers, (*R*)-ketamine and (*S*)-ketamine, in rodents. In addition, (2*R*,6*R*)-hydroxynormetamine [a metabolite of (*R*)-ketamine] and (*S*)-norketamine [a metabolite of (*S*)-ketamine] have been shown to exhibit antidepressant-like effects on rodents through the BDNF–TrkB cascade. In this review, we discuss recent findings on the molecular and cellular mechanisms underlying the antidepressant effects of enantiomers of ketamine and its metabolites. It may be time to reconsider the hypothesis of NMDAR inhibition and the subsequent AMPAR activation in the antidepressant effects of ketamine.

## Introduction

Antidepressants, including selective serotonin reuptake inhibitors (SSRIs) and selective noradrenaline reuptake inhibitors (SNRIs), are widely prescribed for the treatment of depression in patients with major depressive disorder (MDD). However, there is a significant time lag of weeks to months for the antidepressant effects of these drugs to be achieved in patients with MDD^1^. In addition, approximately one-third of patients with MDD do not experience satisfactory therapeutic benefits following treatment with SSRIs or SNRIs^1^. Importantly, the delayed onset of these antidepressants is extremely harmful to patients with depression who experience suicidal ideation^2,3^. Therefore, the development of rapid-acting and robust antidepressants is imperative to relieve the symptoms of severe depression and suicidal ideation in patients with MDD or bipolar disorder (BD)^4–12^.

In 2000, Berman et al.^13^ demonstrated that a sub-anesthetic dose (0.5 mg/kg) of ketamine, an *N*-methyl-d-aspartate receptor (NMDAR) antagonist, produced rapid-acting and sustained antidepressant effects in patients with MDD. This is a first double-blind, placebo-controlled study of ketamine in depressed patients^13^. Subsequently, Zarate et al.^14^ replicated the rapid-acting and sustained antidepressant effects of ketamine for patients with treatment-resistant MDD. In addition, ketamine possesses robust antidepressant effects in patients with bipolar depression^15–18^. Ketamine has been shown to alleviate suicidal ideation in patients with treatment-resistant MDD^19–21^. Several meta-analyses revealed that ketamine has robust antidepressant and anti-suicidal ideation effects in depressed patients with treatment-resistant MDD or BD^2,3,22,23^.

The antidepressant effects of ketamine have attracted increasing academic attention due to its effects being rapid-acting and long-lasting effects in treatment-resistant depression^8,12,24^. Although ketamine has a robust antidepressant effect, its side effects may limit its widespread use for the treatment of depression^12,25–31^. Ketamine has detrimental side effects, which include psychotomimetic effects, dissociative effects, and abuse liability; which may be associated with the blockade of NMDAR^25,26,32^. It is known that dissociative symptoms following ketamine infusion are not associated with its clinical benefits^24^, suggesting that NMDAR inhibition may not serve a key role in the antidepressant effects of ketamine. Fava et al.^33^ also reported that there were no statistically significant correlations between Clinician Administered Dissociative States Scale (CADSS) scores 40 min after the ketamine infusion and Hamilton Depression Rating Scale-6 (HAMD-6) scores at day 1 and day 3 in treatment-resistant patients with depression, in contrast to the hypothesis by Luckenbaugh et al.^34^. In addition, brain-imaging findings suggest that reduced subgenual anterior cingulate cortex is implicated in the antidepressant effects of ketamine in humans^35,36^. However, the precise molecular and cellular mechanisms underlying its antidepressant effects remain unclear. In this review article, recent findings on the molecular and cellular mechanisms underlying the antidepressant effects of enantiomers of ketamine and its metabolites are summarized.

## Enantiomers of ketamine

**Fig. 1:**
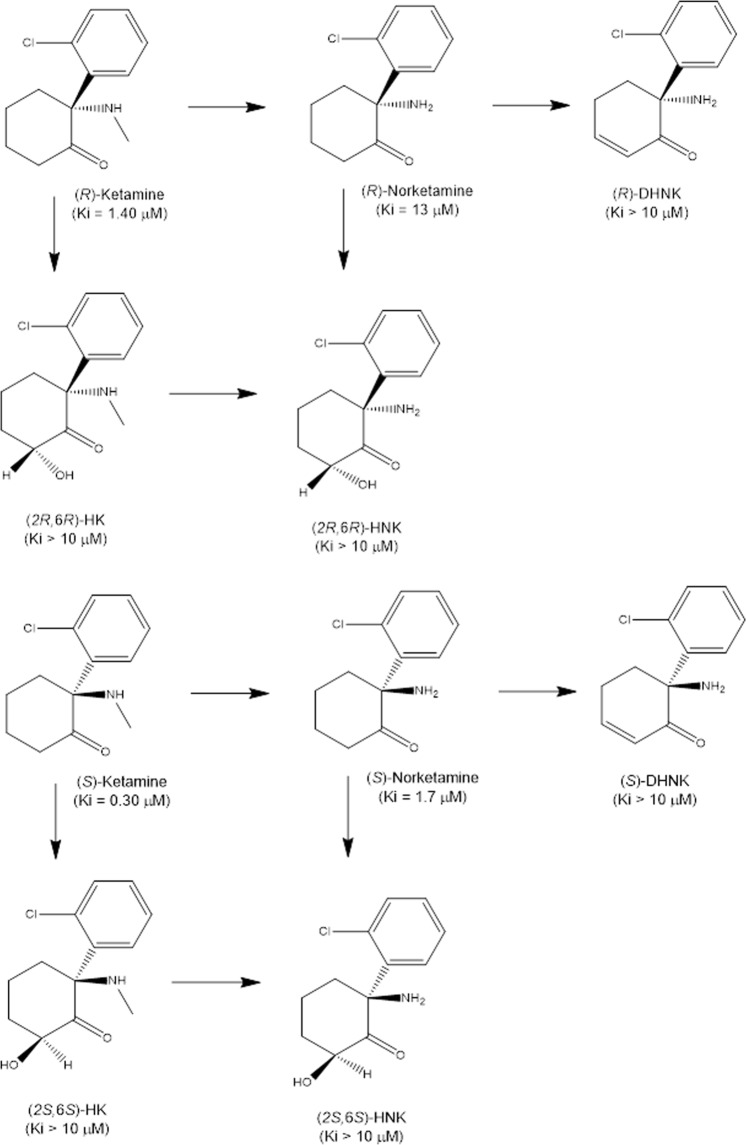
Chemical structure of enantiomers of ketamine and its metabolites. (*R*)-ketamine [or (*S*)-ketamine] is initially metabolized to (*R*)-norketamine [or (*S*)-norketamine] by either CYP2B6 or CYP3A4, and then metabolized to (*R*)-dehydronorketamine (DHNK) [or (*S*)-DHNK]. Hydroxylation of (*R*)-norketamine [or (*S*)-norketamine] at the sixth position by CYP2A6 results in (2*R*,6*R*)-hydroxynorketamine (HNK) [or (2*S*,6*S*)-HNK]. (*R*)-ketamine [or (*S*)-ketamine] is also metabolized to (2*R*,6*R*)-hydroxyketamine (HK) [or (2*S*,6*S*)-HK], then to (2*R*,6*R*)-HNK [or (2*S*,6*S*)-HNK]^49^. The values in the parenthesis are the Ki value for the NMDAR^37,41^

Ketamine (Ki = 0.53 μM for NMDAR) (Fig. 1) is a racemic mixture consisting of equal parts of (*R*)-ketamine (or arketamine) and (*S*)-ketamine (or esketamine). The binding affinity of (*S*)-ketamine (Ki = 0.30 μM) for NMDAR is ~4-fold greater than that of (*R*)-ketamine (Ki = 1.4 μM) (Fig. 1)^37^. Furthermore, the anesthetic potency of (*S*)-ketamine is ~3–4-fold greater and the undesirable psychotomimetic side effects are greater than those of (*R*)-ketamine^38^. We reported that (*R*)-ketamine has more potent and longer-lasting antidepressant-like effects than (*S*)-ketamine in neonatal dexamethasone-treated, chronic social defeat stress (CSDS), and learned helplessness (LH) models of depression^39,40^. Subsequent studies have also shown that (*R*)-ketamine has more potent antidepressant-like effects than (*S*)-ketamine in rodents^41,42^. A recent study showed that the order of antidepressant-like effects in a CSDS model following the intranasal administration is (*R*)-ketamine > (*R,S*)-ketamine > (*S*)-ketamine^43^, and that the order of side effects in rodents is (*S*)-ketamine > (*R,S*)-ketamine > (*R*)-ketamine^43^. The side effects of (*R*)-ketamine in rodents were lower than those of (*S*)-ketamine^40,43–45^. A positron emission tomography study showed a marked reduction in dopamine D_2/3_ receptor binding in the conscious monkey striatum following a single intravenous infusion of (*S*)-ketamine but not that of (*R*)-ketamine, suggesting that the (*S*)-ketamine-induced dopamine release may be associated with acute psychotomimetic and dissociative side effects in humans^46^.

In 1995, Mathisen et al.^47^ reported that the incidence of psychotomimetic side effects of (*S*)-ketamine in patients with orofacial pain was higher than that of (*R*)-ketamine, despite the dose of (*S*)-ketamine (0.45 mg/kg) being lower than that of (*R*)-ketamine (1.8 mg/kg). In addition, Vollenweider et al.^48^ reported that (*R*)-ketamine did not produce psychotic symptoms in healthy subjects and that the majority experienced a state of relaxation, whereas the same dose of (*S*)-ketamine caused psychotic reactions including depersonalization and hallucinations. These findings suggest that (*S*)-ketamine contributes to the acute side effects of ketamine, whereas (*R*)-ketamine may not be associated with these side effects^49^. Importantly, non-ketamine NMDAR antagonists (i.e., memantine, traxoprodil, lanicemine, rapastinel, and AV-101) did not exhibit robust ketamine-like antidepressant effects in patients with MDD^12,22,23^. These clinical findings suggest that NMDAR may not be the primary target for the antidepressant effects of ketamine.

Taken together, (*R*)-ketamine is considered to be a safer antidepressant than (*R,S*)-ketamine and (*S*)-ketamine in humans^12,50–52^. On March 5, 2019, the US Food Drug Administration (FDA) approved (*S*)-ketamine nasal spray for treatment-resistant depression. However, it is only available through a restricted distribution system, under a Risk Evaluation and Mitigation Strategy due to the risk of serious side adverse outcomes. A clinical trial of (*R*)-ketamine in humans is currently underway by Perception Neuroscience, Inc.^12^.

### Mechanisms of action of ketamine’s antidepressant action

#### NMDAR inhibition and subsequent AMPAR activation

**Fig. 2:**
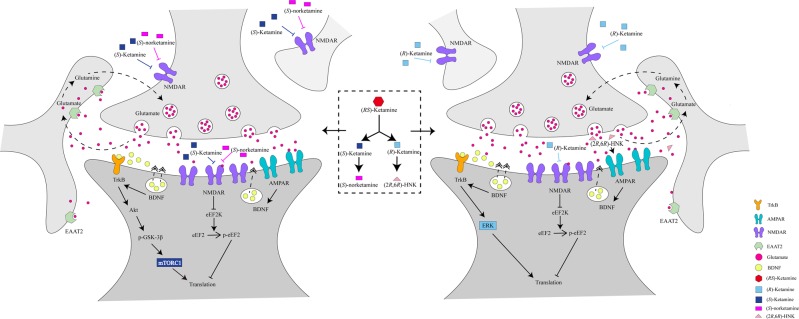
Proposed cellular mechanisms of antidepressant actions of enantiomers of ketamine, and its metabolites. Left: (*S*)-Ketamine is metabolized to (*S*)-norketamine. (*S*)-Ketamine activate AMPAR, subsequently, (*S*)-ketamine activates mTORC1 signaling, resulting in activation of BDNF–TrkB signaling^40,72^. Although (*S*)-norketamine does not activate AMPAR, (*S*)-norketamine activates mTORC1 signaling, resulting in activation of BDNF–TrkB signaling^119^. Right: (*R*)-Ketamine is metabolized to (2*R*,6*R*)-HNK. Antidepressant-like effects of (*R*)-ketamine in rodents are more potent than (*S*)-ketamine, and antidepressant-like effects of (2*R*,6*R*)-HNK are inconsistent. (*R*)-Ketamine activates AMPAR, subsequently, (*R*)-ketamine might activate MEK–ERK signaling, resulting in activation of BDNF–TrkB signaling^40,72^. AMPAR activation may be necessary for antidepressant-like actions of (2*R*,6*R*)-HNK^41^. The mTORC1 signaling and BDNF-TrkB signaling may play a role in the antidepressant effects of (*R*)-ketamine^40,72^

In 1990, Skolnick’ group reported antidepressant-like effects of NMDAR antagonists in rodents^53,54^. Although the precise mechanisms underlying the antidepressant effects of ketamine and its metabolites remain unclear, their rapid antidepressant effects are considered to occur via the blockade of NMDARs located on inhibitory interneurons (Fig. 2). This blockage leads to the disinhibition of pyramidal cells, resulting in a burst of glutamatergic transmission. In 2008, Maeng et al.^55^ reported that α-amino-3-hydroxy-5-methyl-4-isoxazolepropionic acid receptor (AMPAR) antagonists blocked the antidepressant-like effects of ketamine in rodents, suggesting a role of AMPAR activation in the antidepressant-like effects of ketamine. It has been suggested that increased glutamate release activates AMPARs, as AMPAR antagonists inhibit the antidepressant-like effects of ketamine and its two enantiomers^40–42,56–58^. Collectively, it appears that AMPAR activation serves an important role in the antidepressant-like effects of ketamine and its enantiomers^5–10,40,58^.

In contrast, non-ketamine NMDAR antagonists did not produce robust ketamine-like antidepressant effects in depressed patients^12,22,23^. In addition, (*R*)-ketamine has more potent antidepressant-like effects in rodents than (*S*)-ketamine, despite (*R*)-ketamine being less potent at NMDAR inhibition than (*S*)-ketamine. A recent functional MRI (fMRI) study in conscious rats demonstrated that, similar to the potent and selective NMDAR antagonist (+)-MK-801 (0.1 mg/kg), (*R,S*)-ketamine (10 mg/kg), and (*S*)-ketamine (10 mg/kg) produced a significant positive response in the cortex, nucleus accumbens, and striatum. In contrast, (*R*)-ketamine (10 mg/kg) produced negative response in the several regions^59^. This study suggests that (*R*)-ketamine and (*S*)-ketamine induce completely different fMRI response patterns in rat brain, and that (*S*)-ketamine-induced pattern is similar to (+)-MK-801. Collectively, it is likely that at the antidepressant-like dose (10 mg/kg), (*R*)-ketamine does not produce NMDAR antagonist-like brain activation in the brain.

Therefore, it may be time to reconsider the hypothesis of NMDAR inhibition and the subsequent AMPAR activation in the antidepressant effects of ketamine and its two enantiomers. In addition to NMDA inhibition and AMPAR activation, other important pathways, including mechanistic target of rapamycin (mTOR), the brain-derived neurotrophic factor (BDNF)-tyrosine kinase receptor B (TrkB) pathway, may be involved in the antidepressant-like effects of ketamine, as discussed below.

#### Monoaminergic systems

A recent study using in vivo microdialysis showed that (*R*)-ketamine and (*S*)-ketamine acutely increased serotonin (5-HT: 5-hydroxytryptamine) release in the PFC in a dose-dependent manner, and the effect of (*R*)-ketamine was greater than that of (*S*)-ketamine^60^. In contrast, (*S*)-ketamine caused a robust increase in dopamine release compared with (*R*)-ketamine. Differential effects between (*R*)-ketamine and (*S*)-ketamine were also observed in a LPS-induced model of depression. An AMPAR antagonist NBQX attenuated (*S*)-ketamine-induced, but not (*R*)-ketamine-induced 5-HT release, whereas NBQX blocked DA release induced by both enantiomers. This paper suggests differences between (*R*)-ketamine and (*S*)-ketamine in their abilities to induce prefrontal 5-HT and dopamine^60^. Furthermore, Zhang et al.^61^ reported that 5-HT depletion did not affect the antidepressant-like effects of (*R*)-ketamine in a CSDS model, suggesting that 5-HT does not play a major role in the antidepressant-like effects of (*R*)-ketamine.

A recent study showed that dopamine D_1_ receptor activation in the medial PFC may play a role in the antidepressant-like effects of ketamine^62^. However, Chang et al.^63^ reported that the pretreatment with dopamine D_1_ receptor antagonist did not block the antidepressant-like effects of (*R*)-ketamine in a CSDS model, suggesting that dopamine D_1_ receptors may not play a major role in the antidepressant-like actions of (*R*)-ketamine, consistent with the previous report^64^.

Collectively, it is unlikely that monoamines such as 5-HT and dopamine do not play a key role in the antidepressant-like effects of ketamine and its enantiomers although the monoaminergic system may play a role in their other pharmacological effects. A further detailed study is needed.

#### Mechanistic target of rapamycin complex 1 (mTORC1)

mTOR is an atypical serine/threonine protein kinase consisting of 2549 amino acids belonging to the phosphatidylinositol 3-kinase-related kinase family, which combines with several proteins to form two different complexes, mTORC1 and mTORC2^65^. In addition, the signaling pathway controlled by mTOR can regulate physiological function in the central nervous system, such as neuronal development, synaptic plasticity, memory storage, and cognitive function^66^.

In 2010, Li et al. demonstrated that rapamycin, an mTOR inhibitor, inhibited the antidepressant-like effects of ketamine in rodents, which acted by increasing the number of synaptic proteins and synaptic spine density by rapidly activating the mTORC1-signaling pathway in the medial prefrontal cortex (PFC)^56^. In a forced swimming test, ketamine decreased the immobility time and increased the levels of hippocampal mTOR and BDNF, suggesting that the antidepressant-like effects of ketamine may be associated with increased hippocampal levels of mTOR and BDNF^67^. Furthermore, tramadol, an analgesic agent, enhanced the antidepressant-like effects of ketamine by increasing mTOR levels in the rat hippocampus and mPFC^68^. In addition, ketamine and its metabolites [i.e., norketamine and (2*S*,6*S*)-hydroxynorketamine (HNK)] may produce an antidepressant-like effect by increasing the phosphorylation level of mTOR and its downstream targets^69^. By contrast, rapamycin can cause neurobehavioral changes, including anxiety-like behavior, in rats and can impede the antidepressant-like effects of ketamine^70^. In addition, neuropeptide VGF (non-acronymic) knockdown attenuated the rapid antidepressant-like effects of ketamine by reducing mTOR phosphorylation^71^. In dorsal raphe neurons, ketamine transiently increased the neurotransmission mediated by spontaneous AMPAR via mTOR signaling^72^. Furthermore, activation of mTOR in the PFC was involved in the antidepressant-like effects of ketamine, whereas inhibition of this pathway may protect the brain from oxidative stress or endoplasmic reticulum stress^73,74^. The mood stabilizer lithium, a GSK-3 inhibitor, can indirectly activate mTORC1 signaling, thereby enhancing the antidepressant-like effects of ketamine^75^. In addition, we previously reported that ketamine-induced antidepressant-like effects are associated with the AMPAR-mediated upregulation of mTOR and BDNF in the hippocampus and PFC^76^. Collectively, it is likely that mTORC1 signaling serves an important role in the mechanism underlying the antidepressant-like effects of ketamine.

Although these aforementioned studies support the role of mTORC1 in the antidepressant-like effect of ketamine, inconsistent results have emerged in subsequent studies. Autry et al.^57^ showed that the level of phosphorylated mTOR was not altered in the hippocampus of control and *Bdnf*-knockout mice following acute administration of ketamine, and that the antidepressant-like effects of ketamine in wild-type mice were not affected by rapamycin. In addition, another study showed no significant changes in the levels of phosphorylated mTOR in the hippocampus and prefrontal cortex of mice following administration of ketamine or (2*R*,6*R*)-HNK, whereas the levels of phosphorylated eEF2 and BDNF were significantly increased in the hippocampus following administration of ketamine or (2*R*,6*R*)-HNK^41^. This increase may partially explain the mechanisms underlying the sustainable antidepressant-like effects of ketamine^41^. Of note, we reported that mTORC1 serves a major role in the antidepressant effect of (*S*)-ketamine, but not (*R*)-ketamine, in a CSDS model^77^. The antidepressant effects of (*R*)-ketamine may be mediated by the activation of ERK as pretreatment with SL327 (an ERK inhibitor) inhibited the antidepressant effects of (*R*)-ketamine^77^.

There are few clinical studies reporting the role of mTORC1 in the antidepressant effects of ketamine in depressed patients. Denk et al.^78^ reported the first evidence of increased phosphorylated mTOR protein in the blood from a patient with MDD following a single injection of (*S*)-ketamine. Furthermore, we reported that the plasma levels of phosphorylated mTOR, GSK-3β, and eEF2 were significantly increased following a single injection of ketamine^79^. It is, therefore, of interest to investigate whether (*R*)-ketamine can influence ERK and its phosphorylation in the blood from patients with MDD or BD.

A recent randomized, placebo-controlled clinical study demonstrated that pretreatment with rapamycin did not alter the acute effects of ketamine in patients with treatment-resistant MDD, whereas its combination with ketamine prolonged the antidepressant effect of ketamine and the response rate 2 weeks following treatment^80^. At present, there is no evidence that a low dose of rapamycin can achieve sufficient brain levels to inhibit mTOR. It is also suggested that rapamycin may produce beneficial effects through the inflammatory system in the periphery, although further investigation is required. Taken together, the role of mTORC1 in the antidepressant effect of ketamine in patients with MDD remains contradictory. Further investigation using a larger sample size is required to determine the role of mTORC1 in the antidepressant effects of ketamine and its metabolites in patients with MDD.

## BDNF

Multiple lines of evidence show that BDNF and its receptor TrkB serve a critical role in the pathogenesis of depression and therapeutic mechanisms of antidepressants^81–87^. In 2011, Autry et al.^57^ reported that the rapid-acting antidepressant effects of ketamine depend on the rapid synthesis of BDNF, as ketamine did not elicit antidepressant-like effects in inducible *Bdnf*-knockout mice, indicating a key role of the BDNF–TrkB cascade in the antidepressant effects of ketamine. Subsequent studies have supported the role of the BDNF–TrkB cascade in the antidepressant effects of ketamine^67,76^. In addition, the TrkB inhibitor ANA-12 significantly inhibited the rapid and long-lasting antidepressant effects of (*R*)-ketamine, and (*S*)-ketamine in a CSDS model^40^. Furthermore, (*R*)-ketamine produced more marked beneficial effects on reduced synaptogenesis and the BDNF–TrkB cascade in the PFC and hippocampus (i.e., CA3 and DG) of CSDS-susceptible mice than (*S*)-ketamine^40^. It has also been reported that the regulation of glutamate transporter 1 on astrocytes through the activation of TrkB is involved in the beneficial effects of ketamine on behavioral abnormalities and morphological changes in the hippocampus of chronic unpredictable mild stress (CUMS)-exposed rats^88^. A recent study showed that ketamine restores depression-like phenotypes in CUMS-exposed vulnerable rats by rescuing the dendritic trafficking of *Bdnf* mRNA^89^. In addition, the ketamine-induced regulation of TrkB is independent of HNK^90^. Collectively, it is likely that long-lasting activation of the BDNF–TrkB cascade in the PFC and hippocampus may be implicated in the long-lasting antidepressant effects of ketamine and its enantiomers.

### Synaptogenesis

Preclinical studies have shown that ketamine rapidly induces synaptogenesis and reverses the synaptic deficits caused by chronic stress, resulting in its antidepressant-like effects^56,80–94^. We reported that ketamine and its two enantiomers, improved decreased spine density in the mPFC of CSDS-susceptible mice 7 or 8 days following a single dose^40,95^, suggesting long-lasting effects on synaptogenesis. A recent study using single-cell two-photon calcium imaging in awake mice showed that the effects of ketamine on spine formation in the PFC were slower: spine formation rates were not significantly altered at 3–6 h following a single injection of ketamine, but were markedly altered at 12–24 h^96^. This suggests that dendritic spine formation in the PFC was required for the sustained antidepressant effects of ketamine but not for its acute antidepressant effects. By contrast, Zhang et al.^97^ reported that (*R*)-ketamine rapidly (<3 h) ameliorated the decreased spine density in the medial PFC and hippocampus of CSDS susceptible mice, resulting in its rapid acting antidepressant-like effects in rodents. In addition, a recent study showed that (*S*)-ketamine rapidly (<1 h) reversed dendritic spine deficits in CA1 pyramidal neurons of Flinders Sensitive rats with a depression-like phenotype^98^. Therefore, further investigation of the acute effects of ketamine and its enantiomers in the dendritic spine deficits of rodents with depression-like phenotype is required.

## Opioid system

It is well known that ketamine can interact with opioid receptors. The order of affinity for opioid receptor subtypes is mu > kappa > delta. The binding of (*S*)-ketamine is also known to be ~2–4-fold stronger to mu and kappa receptors than that of (*R*)-ketamine^38,99^. In addition, ketamine has been reported to exert antagonistic effects at both mu and kappa opioid receptors, suggesting that ketamine use does not lead to opioid addiction^99^. Recently, pretreatment with an opiate receptor antagonist naltrexone (50 mg) significantly inhibited the antidepressant and anti-suicidal effects of ketamine, but not its dissociative effects, in patients with treatment-resistant MDD, suggesting that activation of the opioid system is necessary to produce the rapid-acting antidepressant effects of ketamine^100,101^. By contrast, Yoon et al.^102^ demonstrated that pretreatment with naltrexone did not affect the antidepressant effects of ketamine in depressed patients with alcohol use disorder. Furthermore, ketamine had antidepressant efficacy in patients concurrently on high-affinity mu opioid receptor agonists (i.e., buprenorphine, methadone, or naltrexone), suggesting that the chronic use of opioid receptor agonists is not a contraindication for ketamine treatment for depression^103^. Therefore, the role of the opioid system in the antidepressant effects of ketamine is controversial.

Recently, we reported that pretreatment with naltrexone did not inhibit the antidepressant-like effects of ketamine in a CSDS model and inflammation-induced model of depression, suggesting that the opioid system may not serve a role in the antidepressant-like effects of ketamine^104^. However, further clinical trials with a large sample size are required to better understand whether opioid receptor activation is necessary for the antidepressant and anti-suicidal effects of ketamine in patients with MDD and BD.

### (2*R*,6*R*)-hydroxynorketamine (HNK)

Ketamine is metabolized to norketamine via *N*-demethylation by cytochrome P450 (CYP) enzymes in the liver (Fig. 1). Following *N*-demethylation, norketamine is further metabolized to HNKs and dehydronorketamine (DHNK) (Fig. 1)^49^. Several metabolites of HNKs, including (2*R*,6*R*;2*S*,6*S*)-HNK and (2*S*,6*R*; 2*R*,6*S*)-HNK, were detected in human plasma following ketamine infusion^105^.

In 2016, Zanos et al. demonstrated that the generation of (2*R*,6*R*)-HNK (Ki > 10 μM for NMDAR) (Fig. 1) in the body was essential for the antidepressant-like effects of (*R,S*)-ketamine in rodents, and that NMDAR may not be involved in the antidepressant-like effects of (2*R*,6*R*)-HNK^41^. Of note, (2*R*,6*R*)-HNK did not produce detrimental side effects (i.e., hyperlocomotion, pre-pulse inhibition deficits, motor incoordination, and abuse liability) of ketamine in rodents at a high dose^37^. Subsequently, several groups have replicated the antidepressant-like effects of (2*R*,6*R*)-HNK in rodents^106,107^. Furthermore, Lumsden et al.^108^ demonstrated that antidepressant-relevant concentrations of (2*R*,6*R*)-HNK did not inhibit NMDAR function, whereas a high concentration (50 μM) of (2*R*,6*R*)-HNK inhibited NMDAR synaptic function^109^. It is also suggested that the metabotropic glutamate mGlu_2_ receptors are involved in the antidepressant-like effects of (2*R*,6*R*)-HNK as the antidepressant-like effects of (2*R*,6*R*)-HNK were absent in mice lacking the *Grm2* gene, but not the *Grm3* gene^110^. It is currently unknown whether mGlu_2_ receptors play a role in the antidepressant-like effects of (*R*)-ketamine in rodents.

By contrast, our group found that (2*R*,6*R*)-HNK did not exhibit antidepressant-like effects in rodent models of depression, however, its parent compound (*R*)-ketamine exhibited robust antidepressant-like effects in the same models^111–116^. Pretreatment with two CYP inhibitors (ticlopidine hydrochloride and 1-aminobenzotriazole) prior to (*R*)-ketamine (3 mg/kg) injection increased the levels of (*R*)-ketamine in the blood, whereas (2*R*,6*R*)-HNK was not detected in the blood. In the presence of these CYP inhibitors, (*R*)-ketamine (3 mg/kg) exhibited antidepressant-like effects, although the same dose did not exhibit antidepressant-like effects in the absence of CYP inhibitors^117^. In addition, we reported that the direct infusion of (*R*)-ketamine in brain regions produced antidepressant-like effects in a rat LH model, suggesting that (*R*)-ketamine itself, but not its metabolite, produced antidepressant-like effects^118^. These data suggest that the metabolism of (2*R*,6*R*)-HNK from (*R*)-ketamine is not essential for the antidepressant-like effects of (*R*)-ketamine^119,120^. The US FDA approved (*S*)-ketamine, however, (2*R*,6*R*)-HNK is not prepared from (*S*)-ketamine, indicating that (2*R*,6*R*)-HNK is not essential for the antidepressant effects of ketamine^12^. A recent study from Zanos et al.^121^ showed that (*R*)-ketamine may exert antidepressant-like effects partly via conversion to (2*R*,6*R*)-HNK. The conclusion was toned down 3 years after the first publication of (2*R*,6*R*)-HNK^41^.

It has also been demonstrated that (2*R*,6*R*)-HNK exerts antidepressant effects through AMPAR activation as AMPAR antagonist inhibited the antidepressant effects of (2*R*,6*R*)-HNK^41^. By contrast, at a clinically relevant unbound brain concentration (0.01–10 μM), (2*R*,6*R*)-HNK did not bind orthosterically or directly to functionally activated AMPARs^122^. Furthermore, (2*R*,6*R*)-HNK failed to evoke AMPAR-centric changes in any electrophysiological endpoint from adult rodent hippocampal sections^122^. Unfortunately, the AMPAR potentiator Org 26576 did not have antidepressant effects in depressed patients^123^. At present, a clinical trial of TAK-653, an AMPAR potentiator with minimal agonistic effects, is underway in patients with treatment-resistant depression (NCT03312894). Further investigation on the role of AMPAR in the action of enantiomers of ketamine and its metabolites (norketamine and HNK) is required.

A recent study demonstrated that a single injection of (2*R*,6*R*)-HNK (1–10 mg/kg), but not (2*S*,6*S*)-HNK, increased aggressive behaviors through AMPAR-dependent mechanisms in the ventrolateral periaqueductal gray matter^124^. A clinical trial of (2*R*,6*R*)-HNK in humans is currently underway at the National Institute of Mental Health, USA^12^. The aggressive effects of (2*R*,6*R*)-HNK in humans warrant investigation. In addition, it is of interest to compare the antidepressant effects of (*R*)-ketamine and its final metabolite (2*R*,6*R*)-HNK in patients with MDD.

### (S)-Norketamine

(*S*)-Ketamine is metabolized to (*S*)-norketamine [Ki = 1.70 μM for NMDAR] by CYP enzymes (Fig. 1). We reported that (*S*)-norketamine, but not (*R*)-norketamine, exhibits rapid and sustained antidepressant-like effects in CSDS and inflammation models of depression. The potency of the antidepressant-like effects of (*S*)-norketamine is similar to that of its parent compound (*S*)-ketamine, although the antidepressant-like effects of (*S*)-norketamine are less potent than those of (*R*)-ketamine^125^. Unlike (*R,S*)-ketamine and its enantiomers, AMPAR antagonists do not inhibit the antidepressant effects of (*S*)-norketamine, suggesting that AMPAR activation appears to be unnecessary for the antidepressant-like effects of (*S*)-norketamine^125^. Therefore, it is unlikely that a rapid increase in glutamate due to the direct inhibition of NMDARs localized to interneurons is involved in the antidepressant-like effects of (*S*)-norketamine (Fig. 2)^125^. Furthermore, we reported that, similar to (*S*)-ketamine, BDNF-TrkB and mTOR signaling might play a role in the antidepressant-like effects of (*S*)-norketamine in rodents^125^. Interestingly, the side effects of (*S*)-norketamine in rodents are significantly lower than those of (*S*)-ketamine; ketamine-induced side effects may be associated with NMDAR inhibition. Taken together, (*S*)-norketamine appears to be a safer alternative antidepressant without the side effects of (*S*)-ketamine in humans^12,125,126^. Of note, unlike (*S*)-ketamine, (*S*)-norketamine is not a schedule compound.

## Conclusions

The discovery of the antidepressant effects of ketamine in depressed patients was serendipitous^24^. The mechanisms underlying the antidepressant effects of ketamine have been investigated for almost 20 years, however, its precise molecular and cellular mechanisms remain to be fully elucidated. Although NMDAR inhibition is considered to serve a key role in the antidepressant effects of ketamine, clinical data of non-ketamine NMDAR antagonists (i.e., memantine, traxoprodil, lanicemine, rapastinel, and AV-101)^12^ and preclinical data using two ketamine enantiomers suggest that mechanisms other than NMDAR inhibition may be involved in the antidepressant effects of ketamine. For example, a randomized, placebo-controlled study using a large sample demonstrated that lanicemine did not exert antidepressant effects in patients with MDD with a history of inadequate treatment response^127^, supporting a lack of antidepressant-like effects of lanicemine in a CSDS model^128^. On March 6, 2019, Allergan announced phase three results of rapastinel as an adjunctive treatment of MDD. In three acute trials, rapastinel treatment did not produce primary and key secondary endpoints compared with the placebo group. By contrast, rapastinel exerted rapid-acting antidepressant-like effects in a CSDS model although, unlike (*R*)-ketamine, rapastinel did not exhibit long-lasting antidepressant effects^129^. Collectively, non-ketamine NMDAR antagonists did not produce robust ketamine-like antidepressant effects in patients with MDD, although certain NMDAR antagonists may exhibit rapid ketamine-like antidepressant-like effects in rodents. There is no guarantee that preclinical data will translate to humans^130^. At present, the general consensus is that NMDAR inhibition and the subsequent AMPAR activation serve a role in the antidepressant-like effects of ketamine and two enantiomers. However, the precise molecular and cellular mechanisms underlying ketamine’s antidepressant actions are complex^5–10,94,131^. Considering the clinical data and new preclinical data using ketamine enantiomers, it is the time to reconsider the current hypothesis for the antidepressant effects of ketamine. Recently, Heifets and Malenka^130^ suggested a need to conceptualize molecular mechanisms with more nuance than action at a single, broadly distributed glutamate receptor.

A number of researchers have used control stress-naive rodents to investigate the antidepressant-like effects of ketamine and its metabolites. Healthy control subjects showed significant increases in depressive symptoms for up to 1 day following a single ketamine infusion^132^, suggesting that ketamine does not produce antidepressant effects in healthy control subjects. It is also well known that ketamine can produce schizophrenia-like symptoms (i.e., positive symptoms, negative symptoms, cognitive impairment) in healthy control subjects^32,38,133^. Therefore, the use of control naive rodents may contribute to discrepancies in the antidepressant-like effects of ketamine and its metabolite HNK^12,134^. Collectively, rodents with depression-like phenotypes should be used to investigate the antidepressant effects of ketamine and its metabolites, although it is clear that animal models of depression cannot fully represent the complexity of human depression^134,135^.

On March 5, 2019, the US FDA approved (*S*)-ketamine nasal spray (Spravato™) for treatment-resistant depression. The clinical study of (*R*)-ketamine and (2*R*,6*R*)-HNK in humans is currently underway^12^. Therefore, it is of interest to compare the antidepressant effects of (*R*)-ketamine and (*S*)-ketamine [or (2*R*,6*R*)-HNK] in patients with MDD or BD. Finally, the identification of novel molecular and cellular targets responsible for the rapid and sustained antidepressant effects of enantiomers of ketamine and its metabolites is useful for the development of novel antidepressants without the detrimental side effects of ketamine.
